# Traumatische Hemipelvektomie: Ein nichtalltäglicher Fall in der Unfallchirurgie

**DOI:** 10.1007/s00113-024-01455-9

**Published:** 2024-07-11

**Authors:** F. Pfalzgraf, M. Ecker, Y. Goßlau, E. Mayr

**Affiliations:** 1 Klinik für Unfallchirurgie, Orthopädie, Plastische und Handchirurgie, Universitätsklinikum Augsburg, Stenglinstraße 2, 86156 Augsburg, Deutschland; 2 Klinik für Gefäßchirurgie und endovaskuläre Chirurgie, Universitätsklinikum Augsburg, Augsburg, Deutschland

**Keywords:** Komplexes Beckentrauma, Hämorrhagischer Schock, Traumatische Koagulopathie, Multiorganversagen, Gefäßrekonstruktion, Complex pelvic injury, Hemorrhagic shock, Traumatic coagulopathy, Multiorgan failure, Vascular reconstruction

## Abstract

Die Herausforderung in der Behandlung der traumatischen Hemipelvektomie ist die Dynamik der komplexen und lebensbedrohlichen Verletzungsfolgen. Dies umfasst zum einen den Haut- und Weichteildefekt, die ossären, nervalen und vasalen Verletzungen, zum anderen die konsekutiv hämostaseologische Entgleisung und Organdysfunktion im Rahmen des Schockgeschehens. Die Behandlung an sich fordert schnelle und zielgerichtete Entscheidungen, um das Leben des Patienten zu erhalten. Im vorliegenden Fall wurde ein 34-jähriger Landwirt zwischen einem Radlader und einem stehenden Anhänger eingeklemmt. Bei seiner Ankunft im Krankenhaus befand sich der Patient in einem hämorrhagischen Schock mit begleitender akuter traumatischer Koagulopathie und einem III-gradig offenen Beckentrauma mit einer vollständigen Ischämie des linken Beins sowie auch einer Blasenverletzung. Nach notfallmäßiger operativer Versorgung und zweizeitiger Stabilisierung des Beckens kam es im weiteren Verlauf zu einer Verschlechterung des Zustandes hin zum Multiorganversagen, wodurch die linksseitige Hemipelvektomie als lebensrettende Maßnahme notwendig wurde. Anschließend waren bei Wundinfektion und bestehenden Haut- und Weichteilschaden mehrere Revisionseingriffe und plastische Rekonstruktionen notwendig. Aufgrund der im Alltag seltenen Konfrontation mit dieser Art von Verletzung und eines nichtallgemeingültigen Therapiealgorithmus soll folgender Fallbericht zum besseren Verständnis der Behandlung sowie auch zur Darstellung der in sich zusammenhängenden Wechselwirkungen der einzelnen betroffenen Organsystemen dienen.

## Abstract (en)

The challenge in treating traumatic hemipelvectomy is the dynamics of the complex and life-threatening consequences of the injury. These include skin and soft tissue defects, osseous, neural and vascular injuries as well as the subsequent hemostatic derangement and organ dysfunction as part of the shock process. The treatment requires rapid and targeted decisions to save the patient’s life. In this particular case a 34-year-old farmer was trapped between a wheeled loader and a stationary trailer. Upon arrival at the hospital the patient was in a state of hemorrhagic shock with accompanying acute traumatic coagulopathy and a grade III open pelvic trauma with complete ischemia of the left leg and a bladder injury. After performing emergency surgery and a two-stage approach for pelvic stabilization the patient’s condition deteriorated up to multiorgan failure, necessitating left-sided hemipelvectomy as an immediate life-saving salvage procedure. In the further course multiple revision surgeries and plastic reconstructions due to wound infections and the presence of skin and soft tissue damage were required. Due to the rare confrontation with this type of injury in everyday practice and the absence of a universal treatment algorithm, the following case report is intended to contribute to a better understanding of the treatment and to illustrate the coherent interactions of the individual organ systems affected.

## Anamnese

Der Landwirt wurde bei Arbeiten auf dem Hof von einem Radlader während des Zurücksetzens erfasst und zwischen dem Heck und einem stehenden Anhänger eingequetscht. Es erfolgte daraufhin die Verständigung des Notarztes. Bei Ankunft des Notarztes war der Patient wach und ansprechbar (Glasgow-Coma-Scale-Wert 15). Der Patient wurde noch am Unfallort intubiert und mit dem Rettungshubschrauber in die Klinik transportiert. Es bestehen weder Vorerkrankungen noch eine Dauermedikation.

## Befund

Bei Ankunft des Patienten in unserer Klinik zeigte sich dieser in der klinischen Untersuchung bei stabilem Thorax intubiert und suffizient beatmet mit einem Punktwert von 3 auf der Glasgow Coma Scale. Der Patient befand sich im Zustand des hämorrhagischen Schocks. In der speziellen Gerinnungsdiagnostik aus dem Schockraum ist bereits eine akute traumatische Koagulopathie mit verlängerter Gerinnungszeit sowie auch einer verminderten Festigkeit des Gerinnsels ersichtlich. Inspektorisch war bei angelegtem Beckengurt ein III°-offenes komplexes Beckentrauma mit aktiver Blutung sowie ausgeprägtem Décollement der linken Flanke von sakroiliakal bis paraumbilikal festzustellen. Auffallend sind eine Pulslosigkeit und ein blasses Hautkolorit der linken unteren Extremität. Die Durchblutung der übrigen Extremitäten war intakt. In dem umgehend durchgeführten Ganzkörper-CT mit Kontrastmittel (Siemens Somatom, Fa. Siemens Healthineers AG, München, Deutschland) zeigten sich eine vollständige Dissoziation des linken Iliosakralgelenks sowie eine Sprengung der Symphyse.

**Abb. 1:**
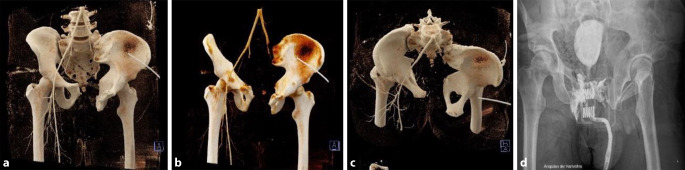
3D-Rekonstruktionen (DeepUnity Diagnost, Fa. Dedalus Healthcare Group, Mailand, Italien) des knöchernen Beckens (a.-p.- und axiale Aufnahme) (**a**–**c**), des Gefäßsystems sowie eine a. p.- Becken-Röntgenaufnahme (DigitalDiagnost, Fa. Philips, Amsterdam, Niederlande) des diffusen Kontrastmittelaustritts aus der Harnblase (**d**)

Im Bereich der rechten Iliosakralregion waren eine Fraktur in der Zone 2 der Massa lateralis mit Beteiligung der sakralen Foramina 2 und 3 zu sehen, wie auch eine Verbreiterung des iliosakralen Gelenkspalts. Des Weiteren zeigten sich Frakturen des SWK 5, der Processus costales LWK 1–4 links und der zwölften Rippe links. Darüber hinaus waren ein Abbruch des Kontrastmittelsignals im Bereich des linken iliakalen Gefäßbündels und eine Aussackung der rechten A. iliaca externa zu sehen. Die Harnblasenwand ist in ihrer Kontinuität unterbrochen und steht nicht mehr in Verbindung zur Urethra (Abb. 1). In den übrig abgebildeten Körperregionen zeigten sich keine weiteren Traumafolgen.

## Aufnahmediagnosen

1. III°-offene traumatische Hemipelvektomie links mit transiliakaler Sakrumfraktur, Symphysensprengung, Fraktur der Processus costae LWK 1–4 links und Abriss der A. und V. iliaca externa links mit kompletter Ischämie des linken Beins,
2. Os-sacrum-Fraktur rechts mit V. a. Dissektion der A. iliaca externa rechts,
3. Fraktur der Costa 12 links,
4. Harnblasenverletzung mit komplettem Harnröhrenabriss,
5. hämorrhagischer Schock,
6. akute traumatische Koagulopathie.

## Therapie

**Abb. 2:**
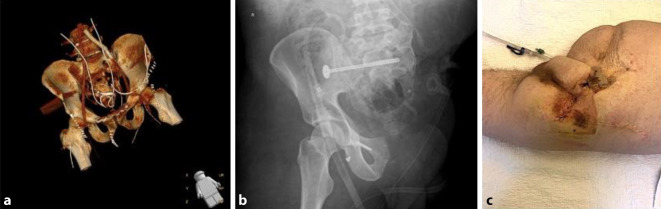
Postoperative 3D Angio-CT Rekonstruktion (DeepUnity Diagnost, Fa. Dedalus Healthcare Group, Mailand, Italien) nach Erstversorgung (**a**) und a.-p-Becken-Röntgenaufnahme (DigitalDiagnost, Fa. Philips, Amsterdam, Niederlande) nach traumatischer Hemipelvektomie (**b**). Situs nach erfolgter Fistelexzision und Hautlappenverschiebeplastik (**c**)

Aufgrund des hämorrhagischen Schocks wie auch der akuten traumatischen Koagulopathie wurde während der Schockraumphase bereits mit der Massentransfusion von Erythrozytenkonzentraten begonnen. Nach Feststellung des Ausmaßes der Verletzung erfolgte umgehend die operative Versorgung. Der Beckengurt war ab Anlage am Unfallort allzeit belassen worden. In der Notfalloperation zeigte sich nach Abnahme des Beckengurts und Inspektion des Situs eine starke arterielle und venöse Blutung, wobei unter Notfall-Packing vorübergehend eine temporäre Bluttrockenheit erreicht werden konnte. Unter Anlage eines Blockadeballons über die Femoralarterie rechts in die linke A. iliaca communis durch die Gefäßchirurgie konnte die knöcherne Situation notfallmäßig stabilisiert werden. Hierzu erfolgten nach offener Reposition die Anlage einer Beckenzwinge sowie eine Symphysenplattenosteosynthese (6-Loch-Platte; Fa. Synthes, West Chester, PA, USA). Damit war es möglich, nahezu anatomische Verhältnisse hinsichtlich des knöchernen Beckens zu erzielen (Abb. 2). Bei Inspektion des Retroperitoneums zeigten sich der sakrale Plexus in der Kontinuität seiner abgehenden Nervenfasern durchtrennt sowie komplette Abrisse der A. und V. iliaca externa links. In gleicher Sitzung erfolgte die arterielle Rekonstruktion der Beckenachse bei längerstreckiger Gefäßverletzung mittels Dacron-Silber-Interponat (Rohrprothese InterGard Knitted Synergy, Fa. Getinge, Getinge, Schweden) von der A. iliaca communis links auf die A. femoralis communis links. Die A. iliaca interna wurde bei Blutung ligiert. Bei verbleibender Dissektion in der A. iliaca communis wurde durch Implantation eines Stent-Grafts (Viabahn Vasc Endo with Heparin, Fa. W.L. Gore & Associates, Newark, DE, USA) von der A. iliaca communis bis in das Interponat die Durchblutung der linken Becken-Bein-Achse wiederhergestellt. Die begleitende linke V. iliaca interna musste ligiert werden. Bei längerer Ischämiezeit des linken Beines wurden die Unterschenkelkompartimente des linken Beines medial und lateral zur Vorbeugung eines Kompartmentsyndroms im Rahmen des drohenden Reperfusionssyndroms gespalten. Die Harnblasenruptur wurde durch die Urologie direkt genäht und mit Anlage zweier Harnleiterschienen sowie einem suprapubischen Katheter versorgt. Die Bauchdecke wurde primär verschlossen. Postoperativ wurde der Patient intubiert und beatmet auf die chirurgische Intensivstation übernommen.

## Verlauf

**Abb. 3:**
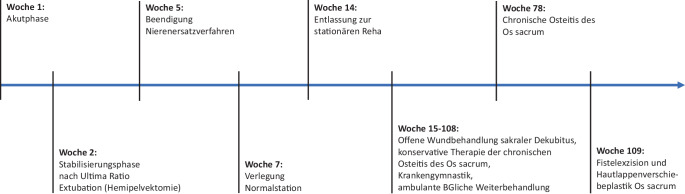
Timeline der Behandlungsmeilensteine

Am Tag des Notfalleingriffs entwickelte sich zudem ein Kompartmentsyndrom des linken Oberschenkels, welches im Rahmen einer zweiten Notfalloperation ebenfalls gespalten wurde. Der Patient war weiterhin kreislaufinstabil und erhielt kumulativ prä-, peri- und am ersten postoperativen Tag 16 Erythrozytenkonzentratbeutel sowie 20 Fresh Frozen Plasma Beutel, 3 Thrombozytenkonzentratbeutel, 2400 I.E. PPSB, 6 g Fibrinogen und 4 g Tranexamsäure, wodurch eine Kompensation des Kreislaufs erreicht werden konnte. Am ersten postoperativen Tag entwickelte sich jedoch im Rahmen des Schockgeschehens sowie auch einer durch Weichteiltrauma/Reperfusionssyndrom bedingten Rhabdomyolyse ein akutes Nierenversagen mit klinisch begleitender Myoglobinurie und laborchemischer Hyperkaliämie mit der Konsequenz einer Hämofiltration. Des Weiteren kam es im Rahmen des hämorrhagischen Schocks zu einem sekundären Leberversagen. Die Gerinnung hingegen konnte am ersten postoperativen Tag stabilisiert werden. Das postoperative Angio-CT (Siemens Somatom, Fa. Siemens Healthineers AG, München, Deutschland) des Beckens zeigte eine Subluxationsstellung des linken und eine Luxationsstellung des rechten Iliosakralgelenkes sowie eine vollständige Durchgängigkeit der implantierten Gefäßgrafts (Abb. 2), sodass wir uns aufgrund des Patientenalters und des kompensierten Zustands des Patienten sowie auch einer perfundierten linken unteren Extremität zur Fortführung der beckenerhaltenden Therapie entschieden. Es folgte somit die komplikationslose beidseitige minimalinvasive ISG-Verschraubung am dritten postoperativen Tag nach offener Reposition (kanülierte Schrauben 7,5 × 115 mm, Fa. AAP Implantate AG, Berlin, Deutschland). Hiernach kam es zu einer zunehmenden Verschlechterung der Perfusion der linken unteren Extremität mit im Verlauf sich entwickelnden Spannungsblasen und beginnenden Nekrosen trotz durchgängiger arterieller Becken-Bein-Achse bei komplettem Verschluss des venösen Abstroms nach Ligatur der V. iliaca externa im Sinne einer Phlegmasia coerulea dolens. Unter progredienter Kreislaufinsuffizienz mit steigenden Katecholaminbedarf kam es zur weiteren Einschränkung der betroffenen Organe, sodass aufgrund des protrahierten Multiorganversagens die notfallmäßige Hemipelvektomie links mit Anlage eines doppelläufigen protektiven Transversostomas am vierten postoperativen Tag nach Erstversorgung durchgeführt wurde (Abb. 2). Nach dem Ultima-Ratio-Eingriff zeigte sich eine rasche Besserung der Organfunktionen. Im Bereich des Operationssitus kam es jedoch zur Superinfektion des postoperativen Hämatoms sowie zu einer ausgeprägten Wundheilungsstörung mit der Folge multipler Revisionseingriffe mit anschließender plastischer Deckung durch Spalthauttransplantationen. In den entnommenen Gewebeproben konnten ein *E. faecalis* als auch *Candida albicans* und *Aspergillus fumigatus* nachgewiesen werden. *E. faecalis* konnte zudem systemisch nachgewiesen werden. Durch die erfolgreiche lokal chirurgische sowie auch systemische antibiotische und fungizide Therapie heilte die Infektion nach mehrwöchiger Therapie aus. Bei Verlegung zur Durchführung der berufsgenossenschaftlichen stationären Weiterbehandlung verblieb nur noch ein chirurgisch nicht zu deckender sakraler Dekubitus, der durch Fortführung einer offenen Wundbehandlung weiter therapiert wurde. Nebenbefundlich entwickelte der Patient während des stationären Aufenthalts einen Hodeninfarkt links mit konsekutiver Orchiektomie, eine Heparin-induzierte Thrombozytopenie des Typs II als auch eine Thrombose der V. jugularis interna beidseits, eine partielle Ulnarisläsion rechts, welche auf das Quetschtrauma zurückzuführen ist, eine Parese des rechten Beines und ein, am ehesten durch die antimykotische Therapie induziertes, Horner-Syndrom. Nach stationärer Rehabilitation wurde der Patient in regelmäßigen Abständen in unserer BG-Sprechstunde betreut. Eine Umwandlung des doppelläufigen Transversostomas in ein endständiges Stoma erfolgte unter Abwägung des Risiko-Nutzen-Verhältnisses bisher nicht. Der sakrale Dekubitus zeigte sich eineinhalb Jahre nach dem Unfall noch nicht endgültig verheilt und superinfiziert mit magnetresonanztomographischem Nachweis einer fokalen Osteitis des Os sacrum unterhalb des sakralen Dekubitus. Nach wiederum testgerechter 6‑wöchiger antibiotischer Therapie konnte die Osteitis erfolgreich behandelt werden. Der sakrale Dekubitus war bis auf eine noch kleine bestehende Fistel mit perifokal instabiler Narbe ebenfalls weiter konsolidiert, sodass nach über zwei Jahren fortwährender Therapie der endgültige Verschluss im Rahmen der Fistelexzision und einer Hautlappenverschiebeplastik erfolgen konnte (Abb. 2). Der Patient ist nun nach fast zweieinviertel Jahren seit dem Traumaereignis weitestgehend schmerzfrei, benötigt jedoch bei noch diskreten Phantomschmerzen eine geringe Dosis an Pregabalin und bei Bedarf Novaminsulfon. Die im Rahmen der rechten ISG-Sprengung bedingte Parese des rechten Beins bildet sich langsam zurück, sodass er sich mit Unterarmgehstützen selbstständig in den Rollstuhl mobilisieren kann. Orthopädietechnisch wurde der Patient mit einer Sitz- und einer Spitzfußorthese versorgt. Zur Verbildlichung des Therapieverlaufs ist dieser in Abb. 3 mit den zwischenzeitlich erreichten Meilensteinen grafisch abgebildet.

## Fallanalyse

Der vorliegende Fall zeigt in seiner Gesamtheit die Komplexität in der Diagnostik, der Therapie und der Fatalität des Verlaufs mitunter mit einhergehender rascher Therapieänderung im Sinne des Ultima-Ratio-Eingriffs als lebensrettende Maßnahme. Diese schwere und komplexe Verletzung wird in der Literatur mit einer Mortalität bis zu 89 % angegeben [1]. Die Ursache für die hohe Wahrscheinlichkeit des Versterbens ist nicht die Fraktur an sich, sondern vielmehr der mit dem komplexen Trauma einhergehende hämorrhagische Schock, die akute traumatischen Koagulopathie, der Weichteilschaden wie auch die Begleitverletzungen der extra- und intraperitoneal gelegenen Organe [2–4].

Im vorliegenden Fall kam der Patient ebenfalls bereits mit einem manifesten hämorrhagischen Schock und einer akuten traumatischen Koagulopathie sowie begleitender Verletzung eines viszeralen Organs und einer Gefäß-Nerven-Verletzung in unserer Klinik an. Ein angepasstes Volumen- und Gerinnungsmanagement prähospital wie auch peri-, intra- und postoperativ sowie die zeitnahe operative Erstversorgung konnten den Patienten trotz der Schwere der Verletzung und der beginnend begleitenden Organdysfunktionen zu einem vorübergehend kompensiert-stabilen Kreislaufzustand verhelfen. Aufgrund des sich zunächst stabilisierten Zustands und des Alters des Patienten wie auch der Reperfusion der linken unteren Extremität, der operativen Versorgung des komplexen Beckentraumas in weniger als 6 h wie auch der Möglichkeit des primären Wundverschlusses und der intakten Anatomie der linken unteren Extremität beruhte die Entscheidung zur extremitäterhaltenden Therapie. Diese Entscheidung musste jedoch kurz nach erfolgter dorsaler Stabilisierung des Beckenrings bei protrahiertem Multiorganversagen und drohendem Exitus letalis revidiert und die verspätete primäre Hemipelvektomie durchgeführt werden. Die Entscheidung gegen die sofortige primäre Hemipelvektomie in Rahmen der operativen Erstversorgung ist in diesen Fall aufgrund der oben genannten in unserer Klinik verwendeten Kriterien und unter Verweis auf vereinzelte monozentrische Studien mit singulärem Nachweis erfolgreicher Erhaltung der Extremität bei warmer Ischämie, zeitnaher Revaskularisierung sowie adäquaten Weichteilverhältnissen und erhaltenem Plexus lumbalis nachvollziehbar. Neben der Kontrolle der Blutung, der Hämostase, der extrapelvinen Begleitverletzungen stellt die Wiederherstellung resp. vorhandene Nervenfunktion ein Hauptkriterium zur extremitätenerhaltenden Therapie dar [5, 6]. Retrospektiv stellt sich die Frage, ob die Hemipelvektomie nicht bereits im Rahmen der ersten Operation hätte erfolgen sollen. Unter Berücksichtigung, dass durch die primäre Hemipelvektomie eine sofortige Stabilisierung der Hämostase ohne Risiko einer postoperativen erneuten Ischämie erreicht werden hätte können, wäre das protrahierte Organversagen möglicherweise vermeidbar und somit die Rekonvaleszenz verkürzt gewesen. Weiterhin spricht für die primäre Hemipelvektomie eine verminderte Inzidenz an Wundinfektionen, bei bereits erhöhten Infektionsrisiko durch die häufig traumabedingten urogenitalen und anorektalen Verletzungen [7]. Somit hätte die Anzahl an Wundrevisionseingriffen und Notwendigkeit der plastischen Deckung durch eine bessere primäre Weichteildeckung trotz sofortiger Behebung der Harnblasenverletzung wie auch der Anlage des doppelläufigen protektiven Transversostomas bei verspäteter primärer Hemipelvektomie möglicherweise vermieden werden können [7, 8]. Zumal spricht für die sofortige primäre Hemipelvektomie die in der Literatur berichteten guten Langzeitergebnisse hinsichtlich verbleibender Phantomschmerzen, einer verminderten Schmerzmitteleinnahme, der besseren Mobilität und einer höheren Patientenzufriedenheit [9]. Bezüglich des postoperativen Verlaufs nach dem Ersteingriff wäre in der Nachschau bei längerer Ischämiezeit des linken Beins und bereits erfolgter Spaltung der Kompartimente der unteren Extremität die primäre Kompartmentspaltung des Oberschenkels unter Vermeidung des zweiten Notfalleingriffs die bessere Verfahrensweise gewesen. Aus unserer Sicht ist mangels hoher Fallzahlen an traumatischen Hemipelvektomien und Fehlen eines standardisierten Therapiealgorithmus die Veröffentlichung der wenigen Einzelfälle zum besseren Verständnis der Komplexität der Verletzungen wie auch als Grundlage zur eigenen Therapieableitung im Falle der Konfrontation mit dieser Art von Verletzung wichtig.

## Fazit für die Praxis

- Bei kritischer Ischämiezeit und angepasst an das neurale und vaskuläre Verletzungsmuster ist primär die Vervollständigung der Hemipelvektomie unter der Doktrin „life before limb“ zu erwägen.
- Die interdisziplinäre Versorgung dieser Art von Verletzung sollte nur in dem nächstgelegenen Krankenhaus der Maximalversorgung erfolgen.
- Es besteht die Notwendigkeit der Festlegung eines standardisierten multizentrischen Therapiealgorithmus.
- Die Ballonblockade der ipsilateralen iliakalen Arterien stellt eine schnelle Möglichkeit zur Kreislaufstabilisierung bei großen arteriellen Verletzungen im Rahmen von Beckentraumata unter Schonung der kontralateralen Beinachse dar.
